# Hesperetin-7-O-Glucuronide Improves Endothelial Cell Function Through Improving NO/ET-1 Balance and Reducing Oxidative Stress via miRNAs

**DOI:** 10.3390/cimb48050538

**Published:** 2026-05-21

**Authors:** Lu Li, Kexin Ji, Fengqi Du, Nini Jin, He Li, Xinqi Liu

**Affiliations:** Key Laboratory of Geriatric Nutrition and Health (Beijing Technology and Business University), Ministry of Education, School of Food and Health, Beijing Technology and Business University, Beijing 100048, China; jkxcc1011@163.com (K.J.); 2230202097@st.btbu.edu.cn (F.D.); 2030202050@st.btbu.edu.cn (N.J.); lihe@btbu.edu.cn (H.L.)

**Keywords:** Hesperetin-7-O-glucuronide, endothelial function, oxidative stress, NO/ET-1, miRNAs

## Abstract

Citrus flavonoid intake is associated with beneficial effects on endothelial function. Our previous randomized control trial demonstrated that the concentration of Hesperetin-7-O-glucuronide (H7G) was positively correlated with the improvement in endothelial function in overweight and obese participants following blood orange juice consumption. To explore the underlying mechanism by which H7G improves endothelial function, we investigated the regulation of H7G on endothelial function in a permanent human endothelial cell line (EA. hy926 cells) under normal and oxidative conditions treated with high-oxidation low-density lipoprotein. The results indicated that H7G improved the expression of nitric oxide synthase 3 (*NOS3*), heme oxygenase 1 (*HMOX1*) ad glutamate cysteine ligase catalytic (*GCLC*), and inhibited the expression of endothelin-1 (*EDN1*), through the upregulation of miR-660-5p and inhibition of miR-21-5p. In summary, H7G improves endothelial cell function via the upregulation of miR-660-5p and the inhibition of miR-21-5p.

## 1. Introduction

Cardiovascular diseases (CVDs) are the leading cause of death worldwide, while posing a serious burden on global economic development [1], accounting for an estimated 32% of all global deaths [2,3]. The coronavirus disease 2019 (COVID-19) that broke out in 2019 has increased the incidence rate and mortality of CVDs [4], urging the need to seek remedy for CVDs. Bioactive substances exert preventive effects against COVID-19 and CVDs without side effects [5,6,7].

Endothelium is a single-layer tissue lining the vascular wall and plays a pivotal role in cardiovascular health, including regulating vascular tone and inflammatory responses. Endothelial dysfunction is manifested by impaired vasodilation function, increased inflammation, elevated oxidative stress, and endothelial cell aging [8,9]. Endothelial dysfunction contributes to the development of diabetes, atherosclerosis and CVDs [10]. Therefore, improving endothelial dysfunction is critical for prevention and treatment of CVDs.

Clinical studies indicate that the consumption of flavonoid-rich orange juice improves endothelial function acutely [11] and chronically [12]. Specifically, citrus flavanones were effective at counteracting the decline in postprandial endothelial function measured as flow-mediated dilation (FMD), potentially through the upregulation of nitric oxide by flavanone metabolites, due to concurrent increases in nitrite and circulating flavanone metabolites [11]. Similarly, our previous randomized control trial indicated that 2-week consumption of blood orange juice increased FMD in adults with overweight and obesity [12]. Notably, the concentration of Hesperetin-7-O-glucuronide (H7G) significantly correlated with FMD, highlighting the critical regulation of endothelial function by H7G. H7G is one of the main flavanone metabolites identified in plasma in adults following orange juice consumption [13]. Similarly, a previous study reported that intravenous administration of H7G exerted hypotensive, vasodilatory and anti-inflammatory activities in hypertensive rats [14]. However, the underlying mechanism of the regulation of endothelial function by H7G has not been studied before.

Endothelial dysfunction is encompassed by complex pathophysiology that is based on endothelial nitric oxide synthase uncoupling and endothelial activation following stimulation from various inflammatory mediators (e.g., molecular patterns, oxidized lipoproteins, and cytokines) [15]. Nitric oxide synthase 3 (*NOS3*) maintains endothelial homeostasis through the nitric monoxide (NO) signaling network, with its function precisely regulated by transcriptional and post-translational modifications, and microenvironmental factors. Uncoupling and epigenetic silencing represent core mechanisms of endothelial dysfunction. Targeting strategies to restore *NOS3* coupling status or gene expression (e.g., demethylation) constitute emerging therapeutic directions for cardiovascular diseases [16]. Endothelin-1 (*EDN1*) is mainly synthesized and secreted by endothelial cells, and regulates endothelial function by activating endothelin receptors, participating in processes such as vascular tension and inflammation [17]. Heme oxygenase-1 (*HMOX1*) exerts multiple protective effects in endothelial function regulation by degrading heme to produce carbon monoxide (CO), biliverdin/bilirubin, and free iron. Its mechanism of action involves antioxidant, anti-inflammatory, anti-apoptotic activities, and maintaining vascular homeostasis [18]. Glutamate cysteine ligase catalytic (*GCLC*) maintains endothelial homeostasis through glutathione-dependent antioxidant systems, anti-iron death, and metabolic reprogramming. Its genetic polymorphism and post-translational modifications (such as succinylation) are key nodes in regulating endothelial function [19]. Reactive oxygen species (ROS) act as signaling molecules to maintain vascular homeostasis at normal physiological concentrations, but when their concentrations are too high, they can cause endothelial dysfunction through oxidative damage, inflammatory activation, and metabolic disorders [20].

miRNAs (microRNAs) are a class of endogenous, single-stranded non-coding RNAs composed of approximately 19–23 nucleotides. They can pair and bind to the 3 ‘UTR region of the target gene, which suppresses the expression level of the target gene after transcription and participates in many cellular reactions [21]. More and more evidence suggests that specific miRNAs can regulate the related functions of endothelial cells; miRNA is a novel molecular target for the treatment of vascular diseases and related diseases such as inflammation [22]. This provides a new perspective for in-depth analysis of the molecular mechanisms by which H7G regulates endothelial function. At present, there is limited research on the regulation of endothelial function by miRNAs mediated by flavonoids, and the mechanism of action remains to be explored [23]. Therefore, this study aimed to explore the regulatory effect of H7G on endothelial cell function under normal and oxidative conditions and the role of miRNAs in this regulation.

## 2. Materials and Methods

### 2.1. Materials and Reagents

Hesperetin-7-O-glucuronide (H7G) was obtained from Toronto Research Chemicals (H288995, purity ≥ 95.0%; Toronto, ON, Canada) and was dissolved in 100% dimethyl sulfoxide (DMSO, LABLEAD, Shanghai, China) at a concentration of 50 μM, then stored at −20 °C. High-oxidation low-density lipoprotein (h-ox) was purchased from Yisheng Biotechnology (20608ES05, Shanghai, China). The control mimic and inhibitor, miRNA-21-5p mimic and inhibitor, and miRNA-660-5p mimic and inhibitor were provided by Guangzhou RiboBio Biotechnology (Guangdong, China).

### 2.2. Cell Culture

Human endothelial cells EA.hy 926 (1101HUM-PUMC000475, BMCR, Beijing, China) were obtained from Peking Union Medical College Cell Bank. Cells were cultured in Dulbecco’s modified Eagle’s medium (DMEM, Gibco, MA, USA) with 1 g/L glucose, 2 mM L-glutamine, 1 mM sodium pyruvate, 10% fetal bovine serum and 0.5% penicillin and streptomycin in a humidified atmosphere with 5% CO_2_ at 37 °C. EA.hy 926 cells were seeded at a density of 1 × 10^5^ cells/mL and left to grow to reach 90% confluence for experiments. Stock solutions of H7G were dissolved in 100% dimethyl sulfoxide (DMSO, LABLEAD, Shanghai, China) at 50 μM in aliquots. Dilutions were made from stock solutions to specific concentrations immediately prior to treatment. Cells were incubated with H7G at concentrations of 0, 1, 5, 10, 25, and 50 μM, with or without h-ox at 25 μg/mL for 24 h.

### 2.3. Determination of Cell Viability

Cells were seeded in 96-well plates at a density of 1 × 10^5^ cells/mL. Following treatment of different solutions for 24 h, cell viability was measured using Cell Count Kit 8 reagent (CCK-8, Biyuntian Biotechnology, Shanghai, China). Absorbance was measured at 450 nm on a multifunctional microplate reader (Infinite 200 Pro Nanoquant, Tecan, Männedorf, ZH, Switzerland). Cell viability was calculated as a percentage relative to the control group (untreated or transfected negative control cells).

### 2.4. Quantitative Real-Time PCR

Total RNA was extracted from EA.hy 926 cells using Trizol reagent (Transgen Biotech, Beijing, China). Reverse transcription of the total cellular RNA was carried out using reverse transcriptase (Transgen Biotech, Beijing, China) with T100 Thermal Cycler (Bio-Rad, Hercules, CA, USA). cDNA samples and specific primers were used to amplify cDNA according to the instructions of the qPCR Quantitation Kit (Transgen Biotech, Beijing, China) through a real-time PCR machine (CFX96 Optics Module, Bio-Rad, Hercules, CA, USA), with ACTB as an endogenous control gene [24]. Reverse transcription of the total cellular miRNA was carried out using reverse transcriptase (Ruibo Biotechnology, Guangzhou, China) via T100 Thermal Cycler (Bio-Rad, Hercules, CA, USA). cDNA samples and specific primers (Ruibo Biotechnology, Guangzhou, China) were used to amplify cDNA according to the instructions of the miRNA qRT-PCR Starter Kit (Ruibo Biotechnology, Guangzhou, China) via a real-time PCR machine (CFX96 Optics Module, Bio-Rad, Hercules, CA, USA), with SnRNA U6 as an endogenous control [25]. The primer sequences of the target genes are shown in Table 1. Data were analyzed by the 2^−ΔΔCt^ method.

### 2.5. Determination of Intracellular SOD, MDA, and NO

Triton X-100 Solution (ST797, Beyotime, Shanghai, China) was used to lyse cells. Concentrations of malondialdehyde (MDA), superoxide dismutase (SOD), and NO in cell lysates were determined using the SOD, MDA, and NO ELISA kits of Nanjing Jiancheng Bioengineering Institute (Nanjing, China). Absorbance was measured at 550, 532, and 450 nm, respectively, using a microplate reader (Infinite 200 Pro Nanoquant, Tecan, Switzerland).

### 2.6. Determination of Intracellular ROS

The ROS content was determined by Fluorescent Probe DCFH-DA as described previously [26]. After gently stripping the cells from the plate and spinning at 4500 rpm for 5 min, the supernatants were transferred and fluorescence intensity was measured at 488/525 nm using a microplate reader (Infinite 200 Pro Nanoquant, Tecan, Switzerland). ROS levels in the cells of each group were measured by fluorescence photography using an inverted fluorescence microscope (IX73, Olympus, Tokyo, Japan). All the data were normalized to protein concentration.

### 2.7. miRNA Sequencing Analysis

EA.hy 926 cells with or without H7G incubation (25 μM, 24 h) were sent for sequencing performed at Shanghai Majorbio Bio-Pharm Biotechnology Co., Ltd. (Shanghai, China). Total RNA was extracted by cell RNA Purification Reagent as per the supplier instructions (Invitrogen, Carlsbad, CA, USA), and the genomic DNA was isolated by rDNase I RNase free (Takara). The quality of RNA was confirmed by a 2100 Bioanalyser (Agilent Technologies, Santa Clara, CA, USA) and further checked by ND-2000 (NanoDrop Technologies, Wilmington, DE, USA). The miRNA expression profiles were performed using the Illumina Hiseq2000 Sequencing platform at Majobio BioTec. Sequenced data were pretreated to eliminate low-quality reads, reads lacking 3′-adapters, 5′-adapter contaminants, and sequences <18 nt and >32 nt. Prediction of target genes was performed using the PathMatch software (version 28.0), followed by annotation of the target genes in the human genome and NCBI databases.

### 2.8. miRNA Transfection

For transfection, EA.hy 926 cells were seeded into 6-well plates until 30–50% confluence. Subsequently, cells were incubated with the formulated miRNA mimics and inhibitors (RIBIO, Guangdong, China) for 24 h at 37 °C in a 5% CO_2_ incubator. miRNA mimics/NC mimics and inhibitors/NC inhibitors were prepared according to the instructions provided by RIBIO. The use of miRNA negative controls (mimetics and inhibitors) ensures the reliability of experimental results by verifying specificity, providing baseline data, and reducing false positives and false negatives, thereby improving the credibility of the data.

### 2.9. Statistical Analysis

Measurements were performed in triplicate for each treatment and concentration. Results of three independent passages were shown as mean ± standard deviation (SD) and analyzed through Statistical Package for the Social Sciences (SPSS, version 26, IBM Corporation, Armonk, NY, USA). A *t*-test was used for comparison between two groups, and differences among groups were analyzed using one-way analysis of variance (ANOVA). Duncan’s multiple comparison test was conducted to further analyze the differences between each treatment group. *p* < 0.05 was regarded as a statistically significant difference.

## 3. Results

### 3.1. Cell Viability

**Figure 1:**
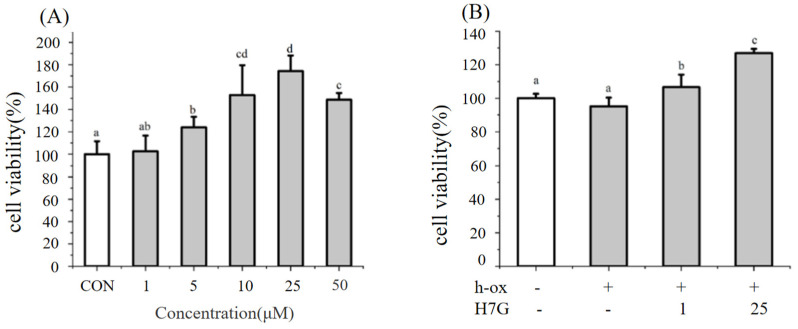
Effect of different concentrations of H7G on the cell viability of EA.hy 926 under normal (**A**) and h-ox (25 μg/mL)-induced oxidative conditions (**B**). Data are presented as the mean value ± SD (biological replicates = 3 and technical replicates = 3). Different letters (a–d) represent significant differences among groups (*p* < 0.05), analyzed by one-way ANOVA followed by Duncan’s multiple comparisons.

In order to investigate the effect of H7G on h-ox-induced oxidative damage in endothelial cells, the CCK-8 method was used to detect cell viability. H7G (5, 10, 25, and 50 μM) significantly improved the cell viability of EA.hy 926 cells (Figure 1A). Subsequently, the effect of H7G at 1 and 25 μM was further investigated under the oxidative condition induced by h-ox (25 μg/mL). Under the oxidative condition induced by h-ox, H7G (1 and 25 μM) increased cell viability in a dose-dependent manner (Figure 1B). The effect of different concentrations of h-ox (25, 50, 75, and 100 μg/mL) on cell viability is shown in Figure S1. Specifically, h-ox exerts inhibitory effects on endothelial cell proliferation, with significant inhibition starting from 50 μg/mL (*p* < 0.05). Therefore, 25 μg/mL was selected for subsequent experiments to investigate the effects of H7G on endothelial function without causing any damage to cell viability while increasing oxidative stress.

### 3.2. Effect of H7G on NO/ET-1

**Figure 2:**
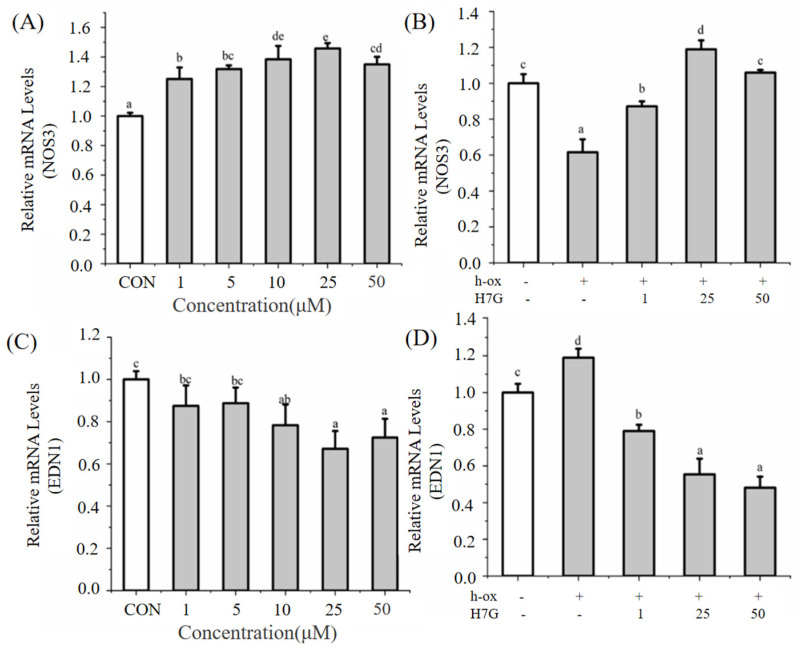
The effect of H7G on *NOS3* mRNA levels under normal (**A**) and h-ox (25 μg/mL)-induced oxidative conditions (**B**); the effect of H7G on *EDN1* mRNA levels under normal (**C**) and h-ox (25 μg/mL)-induced oxidative conditions (**D**).Data are presented as the mean value ± SD (biological replicates = 3 and technical replicates = 3). Different letters (a–e) represent significant differences among groups (*p* < 0.05), analyzed by one-way ANOVA followed by Duncan’s multiple comparisons.

Under normal conditions, H7G (10, 25, and 50 μM) significantly increased the gene expression of *NOS3* compared to the blank control group (Figure 2A). Incubation with h-ox (25 μg/mL) significantly reduced the gene expression of *NOS3* whereas the additional incubation with H7G (1, 25, and 50 μM) significantly upregulated the expression of *NOS3* (Figure 2B).

Under normal conditions, H7G (5, 10, 25, and 50 μM) significantly reduced the expression of *EDN1* compared to the blank control group (Figure 2C). Incubation with h-ox (25 μg/mL) significantly increased the expression of *EDN1* whereas the additional incubation with H7G (1, 25, and 50 μM) significantly downregulated the expression of *EDN1* (Figure 2D). As shown in Figure S2, incubation with h-ox (25 μg/mL) significantly increased the expression of *EDN1* whereas the additional incubation with H7G (1, 25, and 50 μM) significantly downregulated the expression of *EDN1*. Given that *EDN1* is a potent vasoconstrictor and H7G can reduce the levels of *EDN1*, this indicates that H7G may have the effect of regulating vasoconstriction and improving endothelial function.

**Figure 3:**
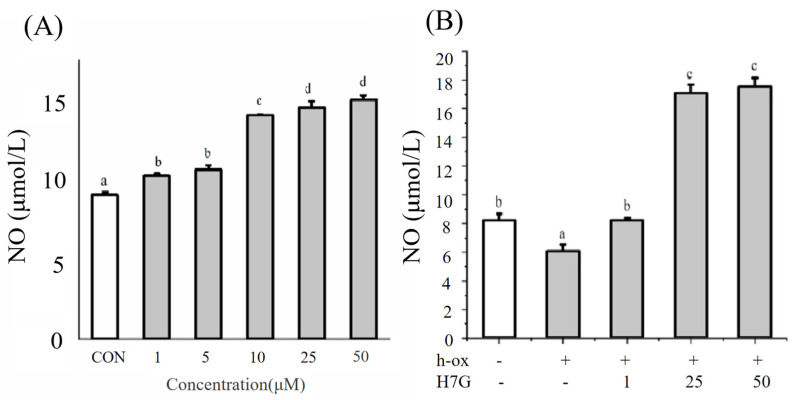
The effect of different concentrations of H7G on NO under normal (**A**) and h-ox (25 μg/mL)-induced oxidative conditions (**B**). Data are presented as the mean value ± SD (biological replicates = 3 and technical replicates = 3).Different letters (a, b, c, and d) represent significant differences among groups (*p* < 0.05), analyzed by one-way ANOVA followed by Duncan’s multiple comparisons.

Under normal conditions, H7G (1, 5, 10, 25, and 50 μM) significantly increased NO compared to the blank control group (Figure 3A). Incubation with h-ox (25 μg/mL) significantly reduced NO whereas the additional incubation with H7G (25 and 50 μM) significantly upregulated NO (Figure 3B).

### 3.3. Effect of H7G on Antioxidant Status-Related Genes and Oxidative Stress

**Figure 4:**
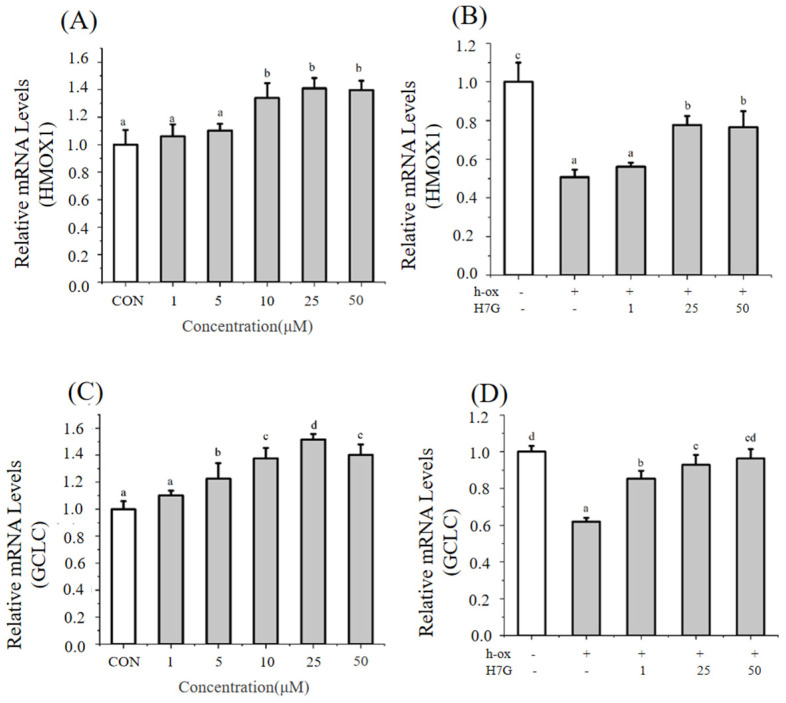
The effect of H7G on *HMOX1* mRNA levels under normal (**A**) and h-ox (25 μg/mL)-induced oxidative conditions (**B**); the effect of H7G on *GCLC* mRNA levels under normal (**C**) and h-ox (25 μg/mL)-induced oxidative conditions (**D**).Data are presented as the mean value ± SD (biological replicates = 3 and technical replicates = 3). Different letters (a–d) represent significant differences among groups (*p* < 0.05), analyzed by one-way ANOVA followed by Duncan’s multiple comparisons.

Under normal conditions, H7G (10, 25, and 50 μM) significantly increased the expression of *HMOX1* compared to the blank control group (Figure 4A). Under normal conditions, H7G (5, 10, 25, and 50 μM) significantly increased the expression of *GCLC* compared to the blank control group (Figure 4C).

Incubation with h-ox (25 μg/mL) significantly reduced the expression of *HMOX1* whereas the additional incubation with H7G (25 and 50 μM) significantly upregulated the expression of *HMOX1* (Figure 4B). Incubation with h-ox (25 μg/mL) significantly reduced the expression of *GCLC* whereas the additional incubation with H7G (1 and 25 μM) significantly upregulated the expression of *GCLC* (Figure 4D).

**Figure 5:**
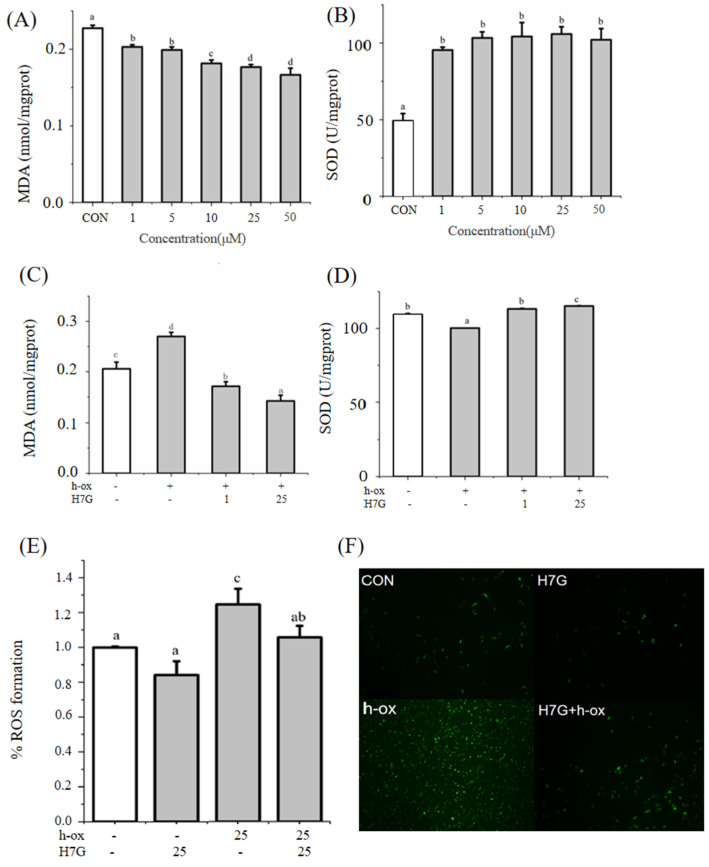
Under normal conditions, the effect of different concentrations of H7G on MDA (**A**) and SOD (**B**); under h-ox (25 μg/mL)-induced oxidative conditions, the effect of different concentrations of H7G on MDA (**C**), SOD (**D**), and ROS (**E**); (**F**) ROS fluorescence staining levels observed under a fluorescence microscope. Data are presented as the mean value ± SD (biological replicates = 3 and technical replicates = 3). Different letters (a–d) represent significant differences among groups (*p* < 0.05), analyzed by one-way ANOVA followed by Duncan’s multiple comparisons.

In order to further investigate the effect of H7G on oxidative stress, the changes in MDA, SOD, and ROS content in endothelial cells were determined. Under normal conditions, H7G (1, 5, 10, 25, and 50 μM) significantly reduced MDA levels (Figure 5A) and increased SOD content (Figure 5B) compared to the blank control group. Compared with incubation with h-ox (25 μg/mL), H7G (1 and 25 μM) significantly reduced the content of MDA (Figure 5C). Incubation with h-ox (25 μg/mL) significantly reduced SOD content whereas the additional incubation with H7G (1 and 25 μM) significantly increased SOD content (Figure 5D).

Under normal conditions, H7G significantly reduced ROS compared to the blank control group, and the ROS content increased following incubation with h-ox. Moreover, under the h-ox-induced oxidative damage condition, the addition of H7G (25 μM) significantly reduced ROS content (Figure 5E).

### 3.4. Regulation of Endothelial Cell Function by H7G via miRNAs

**Figure 6:**
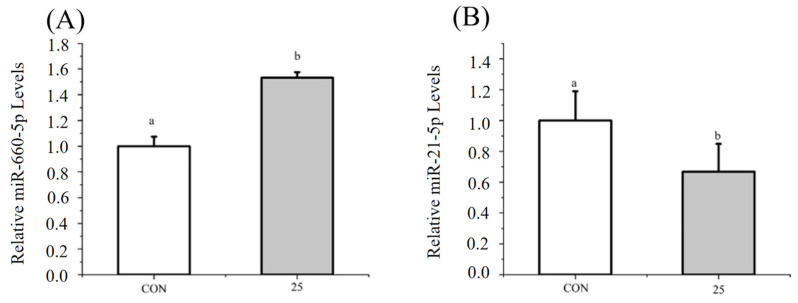
The effect of adding H7G on the levels of miR-660-5p (**A**) and miR-21-5p (**B**). Data are presented as the mean value ± SD (biological replicates = 3 and technical replicates = 3). Different letters (a, b) represent significant differences among groups (*p* < 0.05), analyzed by one-way ANOVA followed by Duncan’s multiple comparisons.

**Figure 7:**
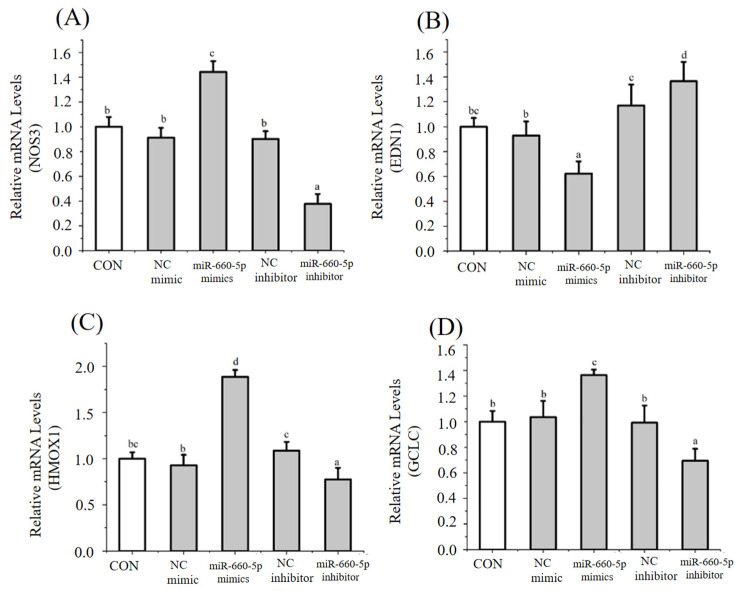
The effect of miR-660-5p mimics on mRNA levels of *NOS3* (**A**), *EDN1* (**B**), *HMOX1* (**C**), and *GCLC* (**D**). Data are presented as the mean value ± SD (biological replicates = 3 and technical replicates = 3). Different letters (a–d) represent significant differences among groups (*p* < 0.05), analyzed by one-way ANOVA followed by Duncan’s multiple comparisons.

**Figure 8:**
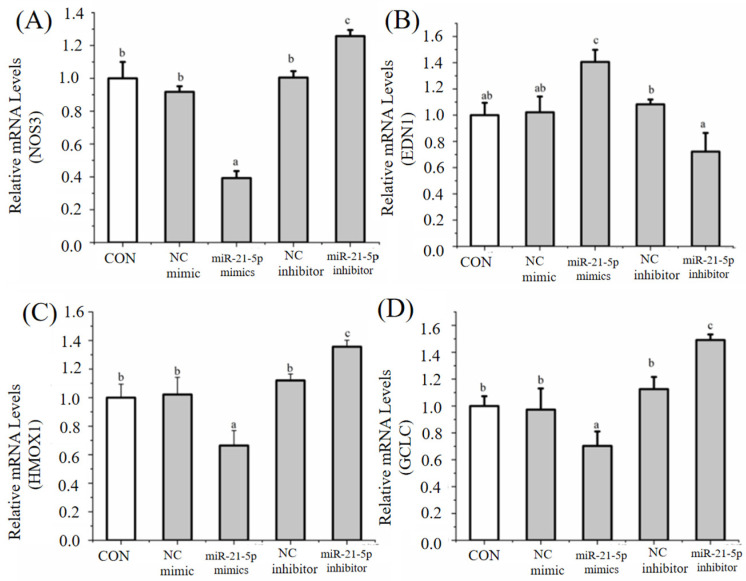
The effect of miR-21-5p mimics on mRNA levels of *NOS3* (**A**), *EDN1* (**B**), *HMOX1* (**C**), and *GCLC* (**D**). Data are presented as the mean value ± SD (biological replicates = 3 and technical replicates = 3). Different letters (a–c) represent significant differences among groups (*p* < 0.05), analyzed by one-way ANOVA followed by Duncan’s multiple comparisons.

Following miRNA sequencing analysis, several miRNAs were selected to further investigate their regulatory effects. Specifically, under normal conditions, H7G (25 μM) significantly reduced the expression level of miR-21a-5p and increased the expression level of miR-660-5p compared to the blank control group (Figure 6A,B). miR-660-5p mimics significantly increased the expression of *NOS3* compared to the blank control group (Figure 7A), while significantly reducing the expression of *EDN1* compared to the blank control group (Figure 7B). Moreover, miR-660-5p mimics significantly increased the expression of *GCLC* and *HMOX1* compared to the blank control group (Figure 7C,D). miR-21a-5p mimics significantly reduced the expression of *NOS3* compared to the blank control group (Figure 8A), while significantly increasing the expression of *EDN1* compared to the blank control group (Figure 8B). Furthermore, miR-21a-5p mimics significantly reduced the expression of *GCLC* and *HMOX1* compared to the blank control group (Figure 8C,D). According to the observation under a bright field and fluorescence, the estimated transfection rate was approximately 85%.

## 4. Discussion

Given that oxidative stress contributes to endothelial dysfunction, which plays a crucial role in the development of CVDs, we have reported for the first time the beneficial effect of the key citrus flavonoid metabolite H7G on NO/ET-1 balance and the expression of antioxidant status-related genes and oxidative stress, while highlighting the key miRNAs involved in this regulation process.

According to our data and the literature, H7G enters cells and regulates gene expression. Specifically, our data demonstrated that H7G improved the expression of *NOS3*, *HMOX1* and *GCLC*, and inhibited the expression of *EDN1*, through the upregulation of miR-660-5p and inhibition of miR-21-5p, while reducing oxidative stress. Similarly, a previous study indicated that H7G entered 3T3-L1 cells, activated peroxisome proliferator-activated receptor-γ (PPARγ), and accelerated adipocyte differentiation, which is also a manifestation of its regulation of gene expression, because PPARγ can regulate the expression of genes related to adipocyte differentiation [27]. In support of these findings, H7G enhanced osteoblast differentiation, and significantly induced the mRNA expression of ALP, Runx2, and osterix, and decreased the gene expression of *RANKL* in primary rat osteoblasts, indicating its role in regulating gene expression [28].

Our results indicated that the intracellular ROS content significantly decreased following the incubation of H7G. According to Figure 5 of the manuscript, H7G (25 μM) significantly reduced the intracellular ROS under oxidative conditions. H7G decreased hydrogen peroxide-induced intracellular adhesion molecule-1 and monocyte chemoattractant protein-1 mRNA expression in rat aortic endothelial cells [14]. Moreover, another study showed that hesperetin glucuronides protected against UV-A-induced necrotic cell death, which may be related to its scavenging of reactive oxygen species [29].

NO produced by endothelial cells can dilate arterial blood vessels, thereby regulating blood pressure and blood flow distribution [30]. A decrease in the bioavailability or insufficient synthesis of NO can lead to NO-dependent endothelial dysfunction, which in turn contributes to the occurrence of various CVDs [31]. Hence, NO plays a crucial role in regulating endothelial function. In this study, H7G significantly increased the level of NO under normal and oxidative conditions through the upregulation of *NOS3*. Similarly, 4-week consumption of orange juice reduced endothelial dysfunction in mild hypercholesterolemic men [32]. *EDN1* is a strong vasoconstrictor that has an antagonistic effect on NO. It can reduce NO secretion by reducing *NOS3* promoter activity and *NOS3* protein levels [33]. In this study, the addition of H7G significantly downregulated *EDN1* expression under normal and oxidative conditions. In agreement with this finding, 12-week consumption of hesperidin-enriched orange juice reduced systolic blood pressure and diastolic blood pressure in mildly hypertensive adults, with a concomitant increase in plasma H7G [34].

Oxidative stress contributes to NO/ET-1 imbalance and endothelial dysfunction [35,36]. ROS are an important indicator of oxidative stress, and when ROS levels increase, it can cause damage to lipids, proteins, and DNA [37]. In this study, H7G significantly increased the levels of SOD and reduced ROS and MDA under normal and oxidative conditions. Similarly, 12-week consumption of orange juice containing a high polyphenol concentration increased SOD activity in overweight or obese adults, with a concomitant increase in hesperetin and naringenin metabolites [38]. In support of this finding, red orange juice supplementation 2.5 h before exercise reduced MDA levels post-exercise in athletes [39]. Nrf2 is known as the first line of defense for cellular antioxidant activity, regulating the expression of various antioxidant-related genes [40]. Research has shown that when Nrf2 translocates to the nucleus, it can activate target genes such as *HMOX1* and *GCLC* for transcription and repair oxidative stress damage [41]. In this study, the addition of H7G significantly upregulated the expression of *HMOX1* and *GCLC* under normal and oxidative conditions. In support of this finding, 12-week consumption of orange juice increased antioxidant capacity and decreased LDL-C and hs-CRP levels in adults with metabolic syndrome [42].

Previous studies have found that miRNAs alleviate endothelial dysfunction through target genes and play an important role in endothelial cell homeostasis [16]. This study indicated that H7G significantly upregulated the level of miR-660-5p and downregulated the level of miR-21-5p. miR-21-5p has been found to regulate oxidative stress, while miR-660-5p can regulate endothelial dysfunction [43,44]. In this study, overexpression of miR-21-5p significantly reduced the levels of *NOS3*, *HMOX1*, and *GCLC*, and increased the levels of *EDN1*. This is consistent with previous research results demonstrating that hypoxia downregulated miR-21-5p, resulting in an increase in the expression of NO-related genes in arterial endothelial cells [45]. In support of this finding, elevated miR-21-5p expression is associated with inflammatory factors such as TNF-α, IL-6 and CRP in hemodialysis patients [46]. Meanwhile, overexpression of miR-660-5p significantly increased the levels of *NOS3*, *HMOX1*, and *GCLC*, and decreased the level of *EDN1*. In support of this finding, miR-660-5p treated breast cancer through downregulating TET2 and activating the PI3K/AKT/mTOR signaling pathway [47]. The PI3K/Akt signaling pathway promotes antioxidant defenses and reduces intracellular ROS [48].

Citrus flavonoids, such as hesperidin and naringin, may play a role in promoting cell survival through various mechanisms in addition to their antioxidant effects. Research has shown that citrus flavonoids can activate the PI3K/Akt signaling pathway, thereby promoting cell proliferation and survival. In addition, they may also affect the activation of MAPK signaling pathways, including ERK and p38 MAPK, thereby promoting cell survival [49]. In addition, studies have shown that citrus flavonoids promote cells to re-enter the proliferative phase from a quiescent state by affecting the regulation of the cell cycle [50]. These findings suggest that the regulatory role of citrus flavonoids in cell survival and related signaling pathways deserves further investigation.

The limitation of this study is the selection of an in vitro model to investigate the effects of H7G on regulating endothelial function, due to the confinement of budget and time. The H7G at 25–50 μM used in this study might have exceeded physiological conditions. The concentration of H7G in the human body can reach the level of μM; after drinking 250 mL of orange juice containing 168 μM hesperidin-7-O-glucoside and 12 μM naringin-7-O-glucoside, the peak concentration of H7G in plasma was 924 ± 224 nmol/L (close to 1 μM) [51], indicating the H7G in plasma can reach the level of μM. In a previous study, osteoblasts were exposed to physiological concentrations of 1 and 10 μM of H7G [28], providing justification for investigating the effects of H7G at 1 and 10 μM via in vitro models. Moreover, our experiment also investigated its effect at 1 μM.

## 5. Conclusions

This study indicates that the key citrus flavonoid metabolite H7G has significant beneficial effects on endothelial cell function. This effect is mainly achieved by regulating the balance between NO/ET-1 and enhancing antioxidant status, under normal physiological conditions and oxidative stress conditions. Notably, H7G further elucidates its potential role in regulating endothelial cell function by upregulating the expression of miR-660-5p and inhibiting the activity of miR-21-5p. These findings provide a new perspective for understanding the role of H7G in cell signal transduction and endothelial function regulation, suggesting its potential application prospects in therapeutic strategies in related fields. Further research is needed to explore the specific mechanisms of action of H7G in different types of vascular diseases and evaluate its potential in clinical treatment.

## Figures and Tables

**Table 1:** Primer sequence.

Gene	Forward Primer	Reverse Primer
*NOS3*	GCAGCCTCACTCCTGTTTTC	GGTCTTCTTCCTGGTGATGC
*HMOX1*	CTTCTTCACCTTCCCCAACA	AGCTCCTGCAACTCCTCAAA
*GCLC*	CAATGGGAAGGAAGGTGTGT	GCGATAAACTCCCTCATCCA
*EDN1*	GATGCCAATGTGCTAGCCAA	GCTGTTTCTCATGGTCTCCG

## Data Availability

The original contributions presented in this study are included in the article. Further inquiries can be directed to the corresponding author.

## Supplementary Materials

The following supporting information can be downloaded at: https://www.mdpi.com/article/10.3390/cimb48050538/s1.

## Abbreviations

The following abbreviations are used in this manuscript:

H7G	hesperetin-7-O-glucuronide
CVD	cardiovascular disease
COVID-19	coronavirus disease 2019
FMD	flow-mediated dilation
h-ox	high-oxidation low-density lipoprotein
DMSO	dimethyl sulfoxide
*NOS3*	nitric oxide synthase 3
*EDN1*	endothelin-1
*HMOX1*	heme oxygenase 1
*GCLC*	glutamate cysteine ligase catalytic
NO	nitric oxide
MDA	malondialdehyde
SOD	superoxide dismutase
ROS	reactive oxygen species
