# Supplementary material for: Hesperetin-7-O-Glucuronide Improves Endothelial Cell Function Through Improving NO/ET-1 Balance and Reducing Oxidative Stress via miRNAs
Source: Curr Issues Mol Biol. 2026 May 21;48(5):538. doi: 10.3390/cimb48050538 (PMC13206419; doi:10.3390/cimb48050538)
Supplement: Supplementary file 1 [file cimb-48-00538-s001.zip › cimb-4264922-supplementary.pdf]

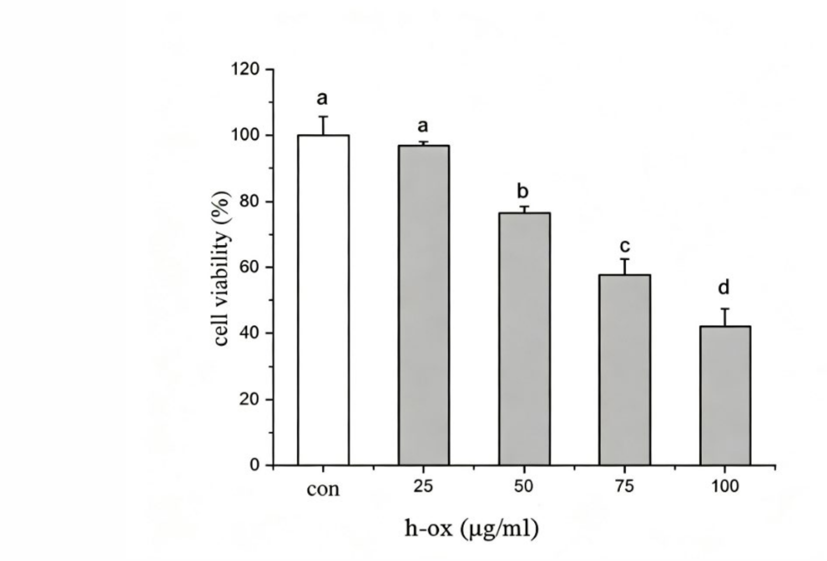

Figure S1 Effects of different concentrations of h-ox on EA.hy926 cell viability.Data were presented as the mean value ± SD (biological replicates = 3 and technical replicates = 3). Different letters (a, b, c, and d) represent significant differences among groups (P < 0.05), analyzed by one-way ANOVA followed by Duncan's multiple comparisons.

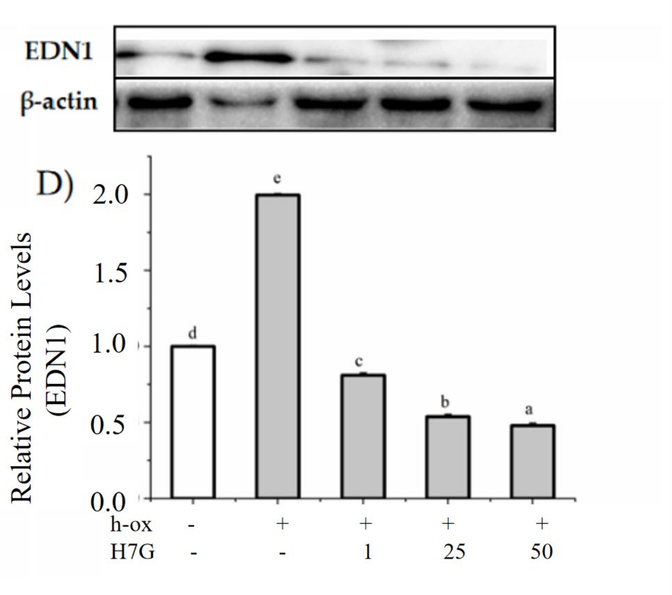

Figure S2 The effect of H7G on ET-1 protein levels under h-ox (25 μg/ml) induced oxidative conditions. Data were presented as the mean value ± SD (biological replicates = 3 and technical replicates = 3). Different letters (a, b, c, and d) represent significant differences among groups (P < 0.05), analyzed by one-way ANOVA followed by Duncan's multiple comparisons.
